# Toward an integrative psychological framework of existential hope: navigating uncertain futures.

**DOI:** 10.3389/fpsyg.2026.1850611

**Published:** 2026-09-10

**Authors:** Ernst Bohlmeijer, Marlon Nieuwenhuis, Alejandro Rodriguez Dominguez, Peter ten Klooster, Catherine Malboeuf-Hurtubise

**Affiliations:** 1 Department of Psychology, Health and Technology, University of Twente, Enschede, Netherlands; 2 School of Psychology, Universite Laval Ecole de Psychologie, Québec City, QC, Canada

**Keywords:** agency, existential hope, existential trust, framework, meaning, uncertainty

## Abstract

Current positive psychological theories conceptualize hope primarily as a cognitive–motivational process, involving beliefs about one’s capacity to generate pathways to desired goals and the perceived agency to pursue them. Such models implicitly rely on a temporality characterized by predictability and control. However, this assumption is not valid for contexts of radical uncertainty in which the future cannot be meaningfully anticipated or is perceived as threatening. This paper articulates an innovative, integrative framework of existential hope informed by a narrative review of the literature that draws on existential, cognitive, and behavioral perspectives. Existential hope is defined as a foundational psychological stance toward life and the future, grounded in existential trust, sustained by a sense of meaning, and expressed through agency – the commitment to act in ways that improve life – even under conditions of uncertainty and perceived threat. In the proposed framework, hope is existential when it is sustained by a belief in the possibility of a good life in the future, even when current developments appear to undermine or contradict such a future. As such, this paper first delineates three core components of existential hope: existential trust, meaning, and agency. It then describes the psychological processes through which individuals may transform experiences of radical uncertainty or existential threat into existential hope, including embracing uncertainty, articulating values, futuring, and relational orienting. Next, the paper discusses how the cultivation of existential hope may contribute to beneficial outcomes, such as increased well-being and civic engagement, alongside reductions in psychological distress. Finally, several pathways for future empirical research are outlined.

## Highlights

- Psychology currently lacks a theoretical framework of hope in the context of radical uncertainty and perceived threat.
- Existential hope is sustained by a belief in the possibility of a good life in the future and a commitment to continued existence, even when current developments appear to undermine or contradict such a future.
- The three core components of existential hope are existential trust, meaning, and agency.
- Psychological processes through which individuals may transform experiences of radical uncertainty or existential threat into existential hope are embracing uncertainty, articulating values, futuring, and relational orienting.
- Cultivation of existential hope may contribute to beneficial outcomes, such as increased well-being and civic engagement, alongside reductions in psychological distress.

## 1 Introduction

Hope is a central construct in positive psychology, closely linked to well-being, resilience, and prosocial behavior (Snyder, 2002; Schornick et al., 2023). Psychological theories typically conceptualize hope as a cognitive–motivational process involving beliefs about one’s capacity to generate pathways toward desired goals and the perceived agency to pursue them (Snyder, 2002). Higher levels of hope are associated with improved academic performance, enhanced problem-solving, better immune functioning, faster recovery from illness, reduced depression risk, greater resilience following trauma, higher relationship satisfaction, and improved workplace functioning (Snyder et al., 2002; Weis and Speridakos, 2011; Chang, 1998; Marques et al., 2017; Gallagher and Lopez, 2009). Hope also correlates positively with prosocial behaviors such as seeking social support, altruism, community involvement, and helping others (Schornick et al., 2023). Within these frameworks, hope functions as a forward-looking expectation of achieving identifiable goals within familiar circumstances and is conceptually related to optimism and self-efficacy (Gallagher and Lopez, 2009).

Other approaches conceptualize hope as an emotion (Miceli and Castelfranchi, 2015; Lazarus, 1999). Edwards et al. (2025), for example, describe hope as a positive affective state related to meaning. This perspective complements cognitive–motivational models by suggesting that hope, as a positive “time-traveling” emotion, provides feedback on goal pursuit and supports long-term striving. Their findings indicate that hopeful feelings indirectly predict meaning in life (Edwards et al., 2025). Beyond Snyder’s cognitive-motivational theory, broader conceptualizations of hope have also been proposed. For example, (Herth, 1992; Nayeri et al., 2020) conceptualized hope as a multidimensional life force encompassing positive future orientation, interconnectedness, inner strength, and meaning. Developed largely in the context of chronic illness and adversity, Herth’s approach highlights relational and existential dimensions of hope that extend beyond goal-directed thinking.

Cognitive approaches rely on a shared assumption: that individuals can envision future states, identify viable pathways, and pursue clearly defined goals (Snyder, 2002). These theories implicitly presuppose a temporal structure characterized by predictability and control. However, this assumption breaks down in contexts where established frameworks of meaning and social order are disrupted and future outcomes cannot be meaningfully anticipated (Malboeuf-Hurtubise et al., 2024). While uncertainty is inherent to hope, existing psychological theories rarely address the role of *perceived uncertainty* or *perceived threat*, despite their growing relevance in contemporary environmental, social, and political conditions.

### 1.1 Hope in the context of radical uncertainty

The concept of uncertainty – particularly *radical* uncertainty – has received considerable attention in philosophy, religious studies, economics, and finance. Radical uncertainty refers to the impossibility of knowlegde-based prediction under conditions of complexity, novelty, or rupture, and has been described as a fundamental feature of the human condition rather than an exceptional state (Arendt, 1958; Sacks, 2012; King and Kay, 2020). Phenomena such as climate change, ecological degradation, political volatility, and socioeconomic precarity do not merely threaten specific outcomes; they undermine the plausibility of stable future horizons (Macy and Johnstone, 2012). Affective responses to ecological crises – such as eco-anxiety – can thus be understood not simply as stress reactions but as systematically generated orientations toward an uncertain future, marked by persistent concern, grief, and moral distress in the absence of actionable solutions (Pihkala, 2020). Similarly, political polarization and declining institutional trust contribute to existential experiences defined by unpredictability and diminished collective coordination (Van Bavel et al., 2024; Mutz, 2024). These developments heighten *perceived uncertainty* and *perceived threat*, shaping how individuals relate to the future. In response, scholars have emphasized the need for existential conceptualizations of hope, including “patient” and “critical” hope (Webb, 2013) and radical hope (Malboeuf-Hurtubise et al., 2024; Ojala, 2023; Damhof and Gulmans, 2023). Unlike conventional “sound hope,” which is tied to specific goals and predictable outcomes, these existential forms of hope are characterized by openness toward the future, willingness to embrace uncertainty, and commitment to sustained engagement despite indeterminate circumstances (Webb, 2013). Yet a comprehensive psychological framework that conceptualizes existential hope – including its core components, underlying processes, and potential outcomes – remains largely absent. A central unresolved question is how individuals sustain engagement, purpose, and action when goals are indeterminate and the future cannot be clearly envisioned.

### 1.2 Study aim

**FIGURE 1:**
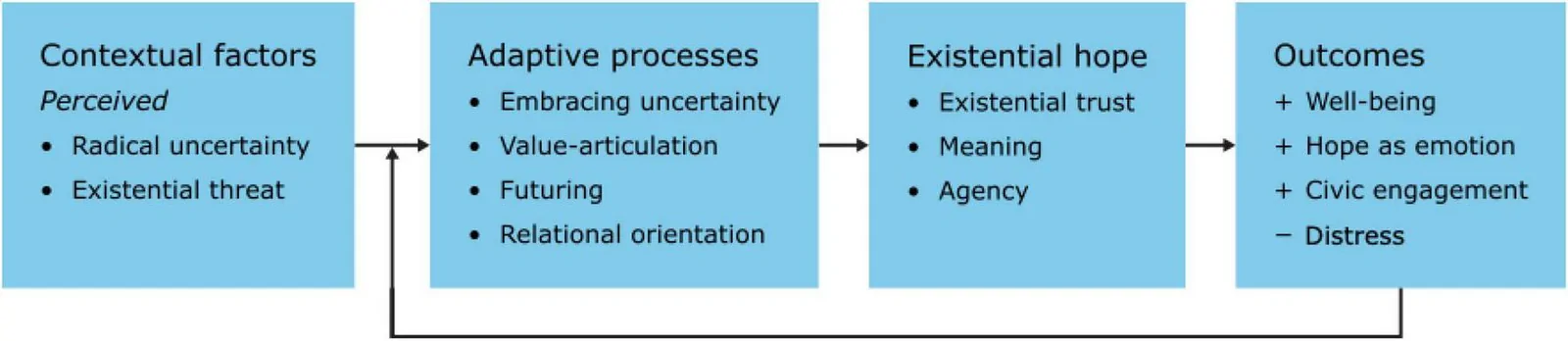
The existential hope framework.

The aim of this paper is to articulate an integrative framework of existential hope informed by a narrative review of existential, cognitive, and behavioral perspectives. This framework is summarized in Figure 1. Existential hope is defined as a foundational psychological stance toward life and the future, grounded in existential trust, sustained by a sense of meaning, and expressed through agency – the commitment to act in ways that improve life – even under conditions of uncertainty and perceived threat. In this framework, hope is existential when it is sustained by a belief in the possibility of a good life in the future, even when current developments appear to undermine or contradict such a future. Existential hope is rooted in courageous imagination: the capacity to envision ways of living a “good life” that transcend present circumstances and perceived reality (Lear, 2006; Frankl, 2006; Damhof and Gulmans, 2023). Following a narrative review of the literature, the paper first examines the core components of existential hope. It then describes the psychological processes through which individuals may transform experiences of radical uncertainty or existential threat into existential hope. Next, it discusses how cultivating existential hope may contribute to beneficial outcomes. Finally, it outlines several pathways for future empirical research.

## 2 Core components of existential hope

Lear (2006) introduced the concept of radical hope in his analysis of the Crow (Apsáalooke) people, whose traditional way of life was devastated by colonial expansion in the 19th century. Radical hope refers to hope for a good life that cannot yet be fully conceptualized – one in which individuals or communities must invent new forms of value and meaning to navigate an unknown future. Unlike conventional hope, it does not rely on precise goals or prior models of success. Instead, it is a forward-looking orientation that persists despite the collapse of familiar structures and the indeterminacy of what a good future may entail.

Existential and humanistic psychologists have similarly emphasized the importance of sustaining meaning and agency under conditions of profound threat. Frankl (2006) argued that even in extreme suffering, individuals can maintain “tragic optimism,” the capacity to pursue meaning and uphold life’s values despite unavoidable hardship. May (1953, 1975) underscored the challenge of courageous engagement with existential uncertainty, emerging when individuals confront threat yet choose to remain committed to life and its possibilities. Yalom (1980) conceptualized psychological distress as rooted in confrontation with ultimate concerns – most centrally death, isolation, freedom, and meaninglessness – and showed how engaging rather than avoiding these givens can promote greater authenticity and vitality. From this perspective, hope is not grounded in the elimination of existential anxiety, but in the capacity to remain engaged with life despite it. Becker (1973) argued that human behavior is shaped by the awareness of mortality, with individuals striving to achieve forms of symbolic immortality through culturally meaningful contributions. In this view, sustaining creativity, and commitment in the face of finitude can be understood as an expression of an existential form of hope. More recently, Wong’s existential positive psychology (PP 2.0) highlights how meaning-focused coping and purposeful engagement enable individuals to thrive in the face of adversity and existential anxiety (Wong, 2020; Wong and Yu, 2021). Although the above authors do not explicitly discuss hope, their emphasis on sustaining agency, meaning, and trust under perceived threat aligns closely with the mechanisms central to existential hope.

In this paper, we use the term existential hope rather than radical hope. Whereas radical hope concerns the preservation of hope when the very concept of the good becomes unintelligible and there is a total collapse of meaning, existential hope refers to the capacity to sustain meaning, agency, and existential trust under conditions in which these are threatened but not yet lost. Drawing on the literature, we propose that existential hope comprises three interrelated components: existential trust, meaning, and agency. Each component is grounded in philosophical, theological, and psychological scholarship, and together they allow existential hope to be operationalized and measured in contexts characterized by perceived uncertainty and perceived threat.

### 2.1 Existential trust

The first core component, existential trust, captures the core feature of existential hope as forward-looking orientation toward a good that cannot yet be fully conceptualized, affirming the possibility of value and a worthwhile future even under conditions of radical uncertainty and perceived threat. Existential trust is not inherently religious or spiritual, although it can be cultivated through religious or spiritual practices. Rather, it is a transcendent stance toward life itself, reflecting a commitment to the potential for a future worth engaging with, even when the content of that future is unknown. Philosophically, existential trust resonates with Tillich’s (1957) notion of ultimate concern, Marcel’s (1961) existential hope, and Frankl’s (2006) logotherapy framework, which emphasizes the human capacity to remain oriented toward value under extreme adversity. Hope is directly related to the experience that life is a journey and that human beings are en route, and that this journey possesses direction and significance (Dauenhauer, 1986; Sacks, 2012; Hasselaar, 2020; Marcel, 1961). Existential trust expresses a confidence in the underlying goodness of the world and a basic sense of existential security (Marcel, 1961; Webb, 2013), embodying the transcendent dimension of hope. As Havel (1991, p. 181) expressed in an interview: “Hope is an orientation of the spirit, an orientation of the heart; it transcends the world that is immediately experienced”. In contrast to conventional hope, which relies on known pathways to goals, existential trust functions as a psychological anchor that sustains orientation toward value and possibility, even when contemporary climate, political and social crises generate deep uncertainty.

Recent research has also highlighted that hope may itself become a source of vulnerability. Harber et al. (2026) introduced the concept of fear of hope, defined as an acquired aversion to feeling hopeful due to the anticipated disappointment or emotional risk that hope may entail. This perspective suggests that individuals may avoid hopeful engagement with the future even when positive possibilities remain available. The concept of fear of hope underscores the importance of existential trust as a willingness to remain open to possibility despite uncertainty and the risk of disappointment.

### 2.2 Meaning

The second component, meaning, reflects the cognitive capacity and processes through which individuals interpret, articulate, and organize experience into a sense of significance. In the context of existential hope, this capacity enables individuals to orient themselves toward the present and near future, even when familiar ways of living or cultural and personal frameworks are threatened. Meaning can arise from confronting what is missing or broken in the world – suffering, inequality, or environmental degradation – which evokes a longing for a different future (Moltmann, 1967; Bloch, 1995). Within existential hope, meaning represents the articulation of a future-oriented longing for what is missing (Webb, 2013). Meaning has a strong narrative dimension arising through the configuration of lived experience across time (Ricoeur, 1992). Hope is sustained through this narrative process by preserving openness to future interpretation and action, even when present circumstances resist coherence (Ricoeur, 1992; Han, 2024). Rather than predicting outcomes, existential hope affirms the possibility of meaning beyond current understanding.

In the psychology field, meaning has been widely studied. For example, Wong (2012) conceptualizes meaning as a multidimensional psychological construct comprising coherence, purpose, and significance, which functions as a value-based cognitive–motivational system that enables resilience and engagement, particularly in the face of suffering and uncertainty. Meaning has also been conceptualized as a narrative process of re-authoring, an ongoing cognitive effort to integrate stressful and distressing experiences into broader belief systems and goals, enabling psychological adjustment (Park, 2010). This perspective is consistent with Janoff-Bulman’s (1992) theory of shattered assumptions, which proposes that traumatic experiences can disrupt individuals’ fundamental beliefs about the world’s benevolence, meaningfulness, and personal worth. Psychological adaptation following trauma often involves reconstructing these disrupted assumptions and developing revised frameworks through which experiences can be understood and integrated. From this perspective, meaning is not merely discovered but actively rebuilt in response to adversity, enabling renewed engagement with life despite uncertainty and loss. Within existential hope meaning is emergent, adaptive, and creative, reflecting the unknown contours of the future. It captures the capacity to orient cognitively toward meaning even when coherence or significance are not yet fully established, allowing for ambiguity and openness.

### 2.3 Agency

The third component, agency, reflects the capacity to act meaningfully and shape one’s future, even under conditions of profound uncertainty and perceived threat, making it a central expression of existential hope. “Action is possible only with a texture of meaning”, writes Han (2024, p. 38). Agency shows the capacity to act intentionally in ways that sustain life, value, and engagement despite uncertainty (Bandura, 2001). Agency in existential hope is not merely the ability to achieve predefined goals; it is the enactment of hope in the face of the unknowable (Bloch, 1995). Sen (1999) conceptualizes agency as the capacity to pursue goals one has reason to value, emphasizing that hope is inseparable from agency because it involves active participation in shaping one’s future rather than passive expectation of outcomes. Hope is sustained not by predicted success but by the freedom to act meaningfully, making agency itself a central expression of hope.

Nussbaum (2011) situates agency within her capabilities approach as the exercise of practical reason and affiliation, that is, the capacity to deliberate and act in ways that are embedded in relationships with others. She argues that human dignity and hope are realized through the capacity to choose and act in pursuit of a life one has reason to value, even under conditions of constraint or uncertainty. According to Nussbaum (2019), hope comprises a vision of a good world and actions aimed at bringing that world about. In the context of existential hope, behavioral expressions of agency might include forms of protest (Moltmann, 1967), acts of care, loving-kindness, and forgiveness (Dauenhauer, 1986; Sacks, 2007; Arendt, 1958), ethical action, or decisions to be patient and await events as a form of positive resignation – a hopeful acceptance of what cannot be forced (Crapanzano, 2003; Marcel, 1961). Agency in existential hope is paradoxical: “It is neither passive waiting nor unrealistic forcing of circumstances that cannot occur” (Fromm, 1968, p. 9). Based on existential trust, hopeful action can have a contemplative dimension, including receiving, waiting, and letting happen (Han, 2024). Existential hope can bring with it “a great mildness, a serene calmness, even a deep friendliness, because it does not enforce anything” (Han, 2024, p. 27). Agency in existential hope functions as the enactment of hope under radical uncertainty and perceived threat, allowing individuals to participate actively in shaping their future and sustaining engagement even when outcomes are unknowable. However, agency within existential hope need not be restricted to individual action or capability; under conditions of pervasive uncertainty it may be expressed through participation in collective action, trust in the actions of others, or engagement with relationships that sustain possibilities for future change.

Together, these dimensions conceptualize existential hope as a dynamic psychological orientation that enables individuals to sustain a sense of possibility, reconstruct meaning, and maintain agency under conditions of profound uncertainty and perceived threat. The content of existential hope may range from personal and immediate concerns to collective and long-term futures; what distinguishes existential hope is not the object hoped for but the encounter with radical uncertainty. Existential trust can be seen as a foundational orientation that precedes cognitive meaning-making and behavioral agency. Without the basic belief that life is worth engaging with, neither meaning nor action can emerge. Meaning serves as the bridge between existential trust and agency: it translates the general orientation toward value into explicit cognitive schemas, interpretations, and commitments that guide potential action. Agency both emanates from and reinforces the other two dimensions: existential trust provides the motivational ground for action, and meaning gives direction and coherence to behavior. Existential hope is conceptualized as emerging from the integration of existential trust, meaning, and agency; while these dimensions may vary in prominence across contexts, no single dimension alone is sufficient to constitute existential hope. This framework provides a basis for understanding how existential hope may emerge and be cultivated in response to radical uncertainty.

## 3 Underlying processes

A second dimension of the framework concerns the psychological processes underlying existential hope. Specifically, the framework addresses the question of which adaptive coping responses enable individuals to transform experiences of radical uncertainty and existential threat into existential hope. Four such adaptive processes are proposed: embracing uncertainty, value articulation, futuring, and relational orienting. In the sections that follow, these processes are discussed and linked to existing psychological concepts and empirical evidence.

### 3.1 Embracing uncertainty

Experiences of radical uncertainty and existential threat are often accompanied by significant psychological distress and discomfort. One illustrative example is eco-anxiety, which refers to the distress individuals may experience when acknowledging or realizing the destructive consequences of human activity on the climate and natural environment (Pihkala, 2020; Hickman et al., 2021). A recent review of 35 studies reported small to large positive associations between eco-anxiety and symptoms of depression and anxiety (Cosh et al., 2024). Manifestations of eco-anxiety may include persistent worry or dread about the future of the planet, low mood, feelings of helplessness or meaninglessness, sleep disturbances, and repetitive negative thinking about environmental collapse. As such, it is closely related to the concept of radical uncertainty. More broadly, radical uncertainty can be understood as a mental state characterized by higher-order metacognition, specifically the conscious awareness of not knowing (Anderson et al., 2019). This awareness distinguishes uncertainty from ignorance. Uncertainty is typically experienced as aversive, and growing evidence suggests that fear of the unknown plays a central role in anxiety disorders and other mental health problems (Carleton, 2016). This relationship may be mediated by processes such as negative mental simulation, whereby individuals imagine adverse or catastrophic future outcomes (Anderson et al., 2019).

A first adaptive response to perceived radical uncertainty and existential threat, therefore, involves the development of tolerance for distress and uncertainty. Distress tolerance has been defined as the capacity to experience and withstand negative emotional states (Simons and Gaher, 2005, p. 83). Empirical studies have consistently demonstrated negative associations between distress tolerance and mental health problems, including depression and anxiety (Laposa et al., 2015; Leyro et al., 2010; Alisson et al., 2020). Individuals with low distress tolerance are more likely to engage in maladaptive coping strategies, which may exacerbate psychological symptoms over time. Closely related is the construct of intolerance of uncertainty, defined as an individual’s tendency to respond to uncertain situations with negative emotions, cognitions, and behaviors (Birrell et al., 2011). A substantial body of research has underscored positive associations between intolerance of uncertainty and psychological distress (e.g., Hill and Hamm, 2019; Rettie and Daniels, 2021; Carleton et al., 2012). From the perspective of existential hope, adaptive responding to uncertainty- and threat-related distress therefore begins with enhancing distress tolerance and reducing rigid, avoidant responses to uncertainty.

Furthermore, acknowledgment and acceptance of uncomfortable emotions induced by perceived uncertainty or threat has consistently been identified as a form of adaptive coping (Lazarus and Folkman, 1984; Ojala, 2012). Within contemporary psychological models, acceptance is conceptualized as a core component of psychological flexibility, enabling individuals to disengage from rigid, maladaptive coping patterns and to persist in value-based behavior even in the presence of distress (Hayes et al., 2012). One promising strategy involves reframing distress as a healthy and proportionate response to genuinely distressing realities, rather than as a symptom to be eliminated (Malboeuf-Hurtubise et al., 2024). The awareness of these concerns can be valued as a token of authenticity and courage (Budziszewska and Jonsson, 2021). Acceptance-based approaches offer practical methods for cultivating a non-judgmental awareness of present-moment experience and for responding to difficult emotions and uncertainty with acceptance rather than avoidance (Hayes et al., 2012; Baer, 2006).

In addition to adaptive emotion-focused coping, individuals could also focus on meaning-focused coping (Ojala, 2012). Meaning-focused coping involves reframing the problem to make it more manageable while taking into account the difficult and supportive emotions that are also experienced. Supportive emotions include gratitude, love, and contentment. They are considered supportive because they can lead to the development of resilience and greater long-term well-being (Hannush, 2021). As such, the concept of meaning-making is closely linked to that of (existential) hope, which is based on an openness to the various possibilities of the future (Hannush, 2021). To promote meaning-focused coping, participants can be invited to explore the challenges and benefits of radical uncertainty in safe spaces such as those that support or therapy groups can provide. Individuals may learn to appreciate certain benefits of uncertainty, such as surprise, openness to new information and possibilities, freedom and the suspension of premature closure (Langer, 2023). It is important to shift from personal attribution to universal attribution of uncertainty. The realization that we are no different in knowing for certain can indeed foster confidence in the presence of uncertainty (Langer, 2023). Within the framework of existential hope, embracing uncertainty does not imply passivity or giving up. Rather, it constitutes an active psychological stance that creates the conditions for subsequent processes, including value and ethics articulation, futuring, and relational engagement. While the relationship between radical uncertainty and existential hope remains to be empirically established, we propose that the capacity to engage with uncertainty in an open and non-avoidant manner may provide a supportive condition for the processes underlying existential hope, including meaning-making, futuring, and relational engagement.

### 3.2 Value-articulation

Embracing radical uncertainty not only alters how individuals relate to distress and the unknown, but also creates the conditions for renewed engagement with values (Lear, 2006). When previously assumed futures, goals, or identities become threatened or untenable, individuals are confronted with the question of what remains meaningful in the absence of certainty or clear instrumental outcomes. Philosophical accounts of existential hope emphasize that, under conditions of profound uncertainty, hope must be grounded in values that can orient action even when the future cannot be fully imagined (Webb, 2013; Dauenhauer, 1986; Han, 2024). In this sense, perceived radical uncertainty and existential threat may function as a catalyst for introspection and value clarification, prompting individuals to articulate what they stand for, care about, and are willing to commit to despite not knowing how circumstances will unfold (Malboeuf-Hurtubise et al., 2024; Ojala, 2017).

From a psychological perspective, values can be understood as emerging from basic human needs and becoming concretized through goals and patterns of action – processes that together constitute motivation. Needs reflect fundamental biological and psychological drivers, whereas values provide higher-order orientation and direction. Schwartz (2017) defines values as desirable, trans-situational goals, varying in importance, that serve as guiding principles in people’s lives. Within Schwartz’s refined theory, nineteen core values are distinguished (Schwartz et al., 2012), which can be broadly organized along a continuum ranging from self-protective, fear-avoidant values to growth-oriented values. Self-protective values emphasize security, control, and continuity, whereas growth-oriented values become more salient when individuals experience relative freedom from threat and fear. Some growth-oriented values primarily focus on personal change and stimulation, while others are inherently social or transcendent in nature, such as care, trustworthiness, concern for nature, and tolerance (Schwartz et al., 2012). These latter values appear particularly congruent with the transcendent orientation characteristic of existential hope.

Increasing awareness of and commitment to values constitutes a central process in Acceptance and Commitment Therapy (ACT), a contemporary cognitive-behavioral approach aimed at enhancing psychological flexibility (Hayes et al., 2012). Psychological flexibility refers to the capacity to remain in contact with difficult internal experiences while persisting in behaviors that are aligned with personally meaningful values. ACT is grounded in the assumption that unwanted sensations, negative emotions, and distressing thoughts are unavoidable aspects of human life, and that attempts to eliminate or avoid them often intensify suffering. Within this framework, values function as a vital motivational compass that supports adaptive action in the presence of uncertainty and discomfort. ACT conceptualizes values as the building blocks of an intrinsically motivating framework for a meaningful life. Values are defined as “freely chosen, verbally constructed consequences of activity patterns, which give these activity patterns meaning and direction and are therefore intrinsically motivating” (Wilson and DuFrene, 2009). Importantly, values are not outcomes to be achieved, but ongoing directions (“paths to be taken”) that are continually revisited, refined, and enacted over the course of life (Hayes et al., 2012). Although values may be translated into concrete goals to facilitate committed action, their open-ended nature aligns closely with the essence of existential hope, which sustains engagement despite the absence of clearly foreseeable outcomes.

### 3.3 Futuring

A third psychological process underlying existential hope is futuring (Damhof and Gulmans, 2023). The importance of future orientation under conditions of uncertainty was already emphasized by Lewin (1942) in his analysis of morale. Lewin argued that individuals’ capacity to maintain commitment and action depends on their ability to connect present circumstances to a meaningful future time perspective. In contexts of disruption and uncertainty, morale is sustained not merely through optimism but through the preservation of valued future possibilities that provide direction for present action. This perspective resonates with existential hope’s emphasis on futuring as a process that enables engagement with uncertain and emerging futures. Futuring refers to the human capacity to engage with the future through expectation, anticipation, and imagination (Melges, 1982). In the context of existential hope, anticipation and imagination are particularly salient. Expectation represents a relatively conservative cognitive process, grounded in prior knowledge and past experience, and therefore tends to reproduce familiar patterns and trajectories. Anticipation, by contrast, opens individuals to possibilities that are not yet realistic or fully imaginable, allowing engagement with unexpected and emergent futures (Sools and Mooren, 2012). The capacity to identify, explore, and navigate multiple possible futures has been conceptualized as *futures literacy* (United Nations Educational, Scientific and Cultural Organization, 2021). Futures literacy supports processes of reimagining what is possible and has been shown to enhance perception by sensitizing individuals to previously overlooked aspects of their social and material worlds (Kazemier et al., 2021). In the context of existential hope, this expanded perceptual field may counteract the narrowing effects of perceived uncertainty and threat. Damhof and Gulmans (2023) emphasize the importance of deliberately stretching the imagination to include futures that initially appear implausible or even preposterous. Engaging with such “impossible” futures can reveal dominant beliefs and narratives about oneself and the world that constrain imagination and reinforce fatalistic or deterministic views. Becoming aware of, and loosening attachment to, limiting and catastrophic narratives about the future – combined with an enhanced capacity to imagine desired alternatives – may induce a renewed sense of agency (Damhof and Gulmans, 2023; Ojala, 2017). Futuring in the context of experienced profound uncertainty and threat differs from imagination as it is commonly understood within Snyder’s (2002) theory of hope. In Snyder’s framework, imagination primarily serves the generation of pathways and the mental representation of routes toward desired, clearly defined goals. Futuring, by contrast, encompasses a broader set of future-oriented capacities, including exploration and engagement with multiple possible futures, even when desired outcomes, pathways, and goals remain uncertain or undefined. Whereas imagination in conventional hope supports movement toward a known future, futuring in existential hope enables engagement with emergent, ambiguous, and fundamentally unknowable futures.

Although desired futures may be experienced initially as images or affective impressions, they are inevitably articulated and elaborated through stories. Human beings make sense of their lives and experiences primarily through narrative, with imagination functioning as a core element of this meaning-making process (Sarbin, 1986; Bruner, 1986). This, in turn, can also nourish adaptive, meaning-making coping strategies. As Polkinghorne (1988, p. 154) noted, “The realization of self as a narrative in process serves to gather what one has been, in order to imagine what one will be, and to judge whether this is what one wants to become.” Building on narrative approaches in psychology, Sools and Mooren (2012) developed the concept of *narrative futuring*, a sense-making methodology that uses storytelling to construct, contest, and rehearse multiple possible futures. Narrative futuring enables individuals and collectives to explore uncertainty, values, and agency in relation to long-term change. A commonly used intervention to elicit narrative futuring is the “letter from the future” exercise (Sools, 2020). In this exercise, individuals are invited to imagine a desired future as if it has already come into being – for example, one in which important wishes have been fulfilled, positive changes have occurred, or adaptive ways of coping with adversity have been developed. Participants then write a letter from this future perspective, describing as concretely as possible what this future looks like and what it is like to live within it.

Futuring and narrative approaches to imagining the future resonate strongly with philosophical accounts of hope under conditions of radical uncertainty, including Jonathan Sacks’ conception of hope as a practice of imaginative moral agency rather than prediction or optimism (Hasselaar, 2020). Sacks (2007) conceptualizes hope as a narrative process through which individuals learn to live with uncertainty while remaining committed to moral action. Within this perspective, identity is understood as the set of images of self and society by which individuals orient themselves in the world. In times of crisis or radical uncertainty, identities grounded primarily in images derived from the past may no longer provide adequate guidance. Under such conditions, individuals are challenged to generate images of a desired future and self and to reformulate their identity in relation to that future-oriented vision. Existential hope, from this standpoint, involves the capacity to trust and act in accordance with a future that cannot yet be fully known or guaranteed. The degree to which individuals are able to cultivate the courage to live by such future-oriented images and the values they embody constitutes a central component of existential hope (Hasselaar, 2020). Sacks (2007) further emphasizes that transforming one’s identity and acting consistently with newly articulated values is a gradual and often tension-filled process that unfolds over time. Sustaining commitment to a desired future, therefore, requires supportive practices and shared rituals that reinforce moral orientation and agency. One such example is the Sabbath, which Sacks describes not primarily as a religious observance, but as a public institution that supports collective reflection and renewed attention to the future one seeks to bring about. In this sense, public forms of Sabbath can function as practices of “utopia now,” offering temporal and symbolic space to sustain courage, motivation, and commitment to values grounded in images of desired futures (Hasselaar, 2020).

Within the framework of existential hope, futuring thus serves as a crucial process through which values are translated into lived possibilities and renewed agency emerges despite perceived uncertainty and threat.

### 3.4 Relational orienting

A fourth underlying process of existential hope is relational orienting. Relational orienting refers to the tendency to navigate an uncertain future through relationships with others rather than as an isolated individual. When people face situations in which outcomes cannot be predicted or controlled, existential hope is often sustained through shared practices, mutual commitment, dialogue, and a sense of belonging. Webb (2013) defines hope as a socially mediated human capacity, emphasizing that its emergence and sustenance are deeply embedded in social contexts. Several scholars similarly consider relational orienting as a vital condition for the emergence of existential hope. In the concept of patient hope, for example, individuals place their trust in a shared journey rather than in specific outcomes (Webb, 2013). Similarly, Dauenhauer (1986) suggests that hope involves seeking transformation in one’s relationships with other humans and with the natural world, and that such relational transformations are intrinsically valued (Webb, 2013).

Relational orienting becomes particularly important in contexts of radical uncertainty. When the future is unknowable, people often rely less on prediction and more on enduring relationships and mutual commitment (Baier, 1986). Social bonds endure not because outcomes are predictable, but because people trust one another’s continued presence and engagement. Lear’s (2006) conceptualization aligns closely with this perspective. Radical hope, in his analysis, is not sustained solely at the level of the isolated individual but is inherently relational and communal. It emerges most clearly when shared cultural meanings, practices, and roles have been disrupted. Under such conditions, hope depends on maintaining relational bonds through which identity, meaning, and moral orientation can be preserved and reworked. In Lear’s account of Plenty Coups, Chief of the Crow Nation, commitment to a future that cannot yet be fully understood is enacted through continued participation in communal practices, storytelling, and ethical leadership. The role of relationships in navigating uncertainty is also consistent with Schachter’s (1959) classic work on anxiety and affiliation. Schachter described that individuals facing ambiguous and threatening situations often seek the company of others as a means of reducing uncertainty and regulating emotional distress. These observations suggest that social connection serves not only supportive functions but also provides a relational context through which individuals make sense of uncertain futures. Such processes align closely with relational orienting as a mechanism through which existential hope is sustained.

Sacks likewise highlights the centrality of relational orienting in cultivating hope under radical uncertainty (Hasselaar, 2020). Two Hebrew concepts are particularly salient: *emunah (faith)* and *chesed (loving-kindness)*. *Emunah* represents a form of relational knowledge guided by empathy, fostering trust and affirmation (Sacks, 2011). *Chesed* reflects a commitment to seeing oneself and others as inherently valuable, expressed through kindness, care, and steadfast loyalty (Sacks, 2007). Similarly, Ojala (2012) emphasizes that trust in others and a sense of belonging to a larger community are important building blocks of hope. Collaborative engagement toward shared goals further strengthens collective agency and efficacy. Existential hope, therefore, is not merely an individual orientation but a communal process. Malboeuf-Hurtubise et al. (2024) describe it as a dynamic through which individuals and communities transform suffering into new forms of well-being. Existential hope engages collective memory while orienting communities toward meaningful change that supports societal flourishing (Ojala, 2023).

Relational orienting is also deeply grounded in psychological theory. The need to belong and to form and maintain strong, meaningful interpersonal relationships is considered a fundamental human motivation (Baumeister and Leary, 1995). There is substantial evidence that relatedness constitutes a basic psychological need that fosters intrinsic motivation, self-determined functioning, civic engagement, and well-being (Ryan and Deci, 2000, 2017). As such, social bonding can be understood as the establishment of a connection based on shared feelings, interests, or experiences (Algoe and Jolink, 2021). Unlike mere liking, bonding is a reciprocal and interactive process that nurtures enduring connection. Brown (2012) describes social connection as the energy between people that arises when individuals feel seen, heard, and valued; when they can give and receive without judgment; and when they derive strength and sustenance from the relationship.

Empirical research identifies several psychological processes that facilitate relational orienting. One such process is self-disclosure (Masaviru, 2016). Social penetration theory conceptualizes bonding as a gradual process in which individuals progressively share emotionally and morally significant aspects of themselves through responsive interaction (Altman and Taylor, 1973). In contexts of distress or radical uncertainty, such interactive self-disclosure can promote a sense of common humanity – the recognition that suffering and struggle are shared aspects of the human condition rather than isolated personal failures (Neff, 2023a). Common humanity may further evolve into active, relational forms of compassion that motivate individuals and communities to take protective and constructive action together, even when doing so involves discomfort or assertiveness. This, however, implies a heightened responsiveness to others.

Responsiveness is widely regarded as a defining feature of relational orienting and high-quality relationships (Reis and Gable, 2015). It encompasses three interrelated components: understanding (awareness of the other’s needs, desires, strengths, and vulnerabilities), validation (respect and appreciation for the other’s experiences), and caring (emotional investment in the other’s well-being). Responsiveness is inherently reciprocal: it involves accurately perceiving another’s needs and responding with warmth, recognition, and support. In addition, the shared experience of positive emotions, the expression of gratitude, and the capacity for forgiveness contribute to resilient and vital relationships (Algoe, 2019; McCullough, 2008; Karremans and Van Lange, 2008).

Within the framework of existential hope, relational orienting appears to serve a dual function. First, it provides a context of psychological safety in which individuals can express distress related to perceived uncertainty and threat, and feel understood and acknowledged. Such relational safety enables exploration of personal values and desired futures. Second, relational orienting supports the formation of partnerships and communities in which individuals commit to collective action based on shared values and envisioned futures. In this way, relational orienting not only sustains hope under radical uncertainty but also translates hope into coordinated moral and civic engagement.

Although presented separately for conceptual clarity, the four processes underlying existential hope – embracing uncertainty, value-articulation, futuring, and relational orienting – are interrelated and mutually reinforcing. Embracing uncertainty creates the psychological space in which defensive avoidance gives way to reflection (Malboeuf-Hurtubise et al., 2024). Within that space, value-articulation provides moral orientation and direction when familiar goals or identities collapse. Futuring then enables individuals and communities to imaginatively explore how these values might take shape in possible worlds that cannot yet be fully anticipated. Relational orienting, finally, offers the social context in which distress can be held, values negotiated, and envisioned futures enacted collectively. Rather than unfolding linearly, these processes form a dynamic and recursive pattern: relationships support the courage to face uncertainty; shared values guide collective imagination; and imagined futures, in turn, strengthen commitment and communal bonds. Existential hope thus emerges not from a single mechanism, but from the ongoing interplay of emotional, moral, imaginative, and relational capacities.

## 4 Outcomes

In our integrative framework, higher levels of existential hope are hypothesized to result in several beneficial outcomes. Key proposed outcomes include emotional hope, psychological well-being, and civic engagement.

### 4.1 Emotional hope

The first outcome concerns the emotional domain. Although there is ongoing debate regarding whether hope should be understood primarily as an emotion or as a cognitive–motivational orientation toward the future, many scholars acknowledge that hope has important affective manifestations. In the present framework, existential hope is conceptualized as a multidimensional orientation comprising existential trust, meaning, and agency, rather than as an emotion *per se*. However, these underlying processes are expected to give rise to hopeful feelings and other positive emotional experiences. Affective hope may be understood as a positive emotion that encourages sustained motivation and perseverance in the face of delayed or uncertain outcomes (Edwards et al., 2024). Research on positive emotions suggests a possible mechanism through which such affective experiences may contribute to well-being. According to the Broaden-and-Build Theory (Fredrickson, 2001; Fredrickson and Brannigan, 2005), positive emotions broaden individuals’ thought–action repertoires, increasing openness, cognitive flexibility, exploration, and social engagement. Over time, these processes can contribute to the development of enduring psychological and social resources. To the extent that existential hope generates hopeful and other positive emotional states, it may similarly contribute to these beneficial processes. However, whether existential hope produces such effects through its affective manifestations, and whether these effects differ from those associated with more conventional forms of hope, remains an important question for future empirical research.

### 4.2 Psychological well-being

A second proposed outcome is psychological well-being. Psychological well-being concerns optimal *functioning* rather than merely *feeling* good. It can be conceptualized as a form of self-actualization (Ryff, 1989), reflecting the extent to which individuals realize their potential. Lear (2006) conceptualized that radical hope brings forth radically different forms of living a virtuous life and excellence. Psychological well-being encompasses dimensions such as purpose in life, personal growth, autonomy, environmental mastery, self-acceptance, and positive relationships. Self-actualization involves accepting oneself, cultivating harmonious and intimate relationships, and autonomously directing one’s life in accordance with personally endorsed values. These processes contribute to personal growth and a coherent life narrative. Substantial research demonstrates that psychological well-being is associated with better physical health, increased resilience, and a reduced risk of mental disorders (e.g., Ryff, 2014; Ten Klooster et al., 2026; Wood and Joseph, 2010). Existential hope, as conceptualized in this framework, involves sustaining meaning and trust under conditions of uncertainty and perceived threat and developing perspectives for action grounded in personal values. Such orientation is closely aligned with the core components of psychological well-being, particularly purpose in life, personal growth, and autonomy. Existential hope may therefore directly lead to psychological well-being by enabling individuals to function coherently and constructively despite existential disruption.

### 4.3 Civic engagement

A third proposed outcome is civic engagement. Civic engagement refers to the ways in which citizens participate in community life to improve social conditions and help shape a shared future (Adler and Goggin, 2005). It includes activities such as community service, collective action and political involvement (Diller, 2001) and in the context of climate change, can include adopting proenvironmental behaviors (Pelletier, 2002). Civic engagement has been associated with improved physical and mental health (Guo et al., 2017), as well as with collective efficacy and bonding social capital, including trust, reciprocity, and shared norms (Collins et al., 2014). Importantly, different forms of hope relate differently to civic engagement. Naïve hope – often characterized by denial or optimistic assumptions that problems will resolve themselves – has been found to be negatively related to civic engagement (Ojala, 2017). In contrast, existential hope may be positively associated with civic engagement. Rather than avoiding distressing realities, we argue that existential hope arises from acknowledging present threats while reflecting on desired futures. This contrast between the current situation and envisioned alternatives may motivate sustained collective action.

## 5 Discussion

In this paper, we have presented an integrative framework of existential hope and outlined its core components. Before turning to future research directions, it is important to clarify the dynamic nature of the proposed relationships.

Although the framework may appear linear – moving from perceived contextual conditions to adaptive processes, existential hope, and outcomes – we conceptualize these relationships as cyclical and mutually reinforcing. Existential hope is not a one-directional pathway but a recursive process characterized by feedback loops. For example, civic engagement and participation in communities of active, value-driven individuals may strengthen relational orienting and enhance feelings of trust, agency, and meaning (Ojala, 2017). Engaging collectively can reaffirm chosen values and deepen commitment to them. In a similar vein, the positive emotions that arise from existential hope – such as hope, gratitude, and love – may broaden behavioral repertoires and foster adaptive coping, social openness, and initiative. These processes may, in turn, reinforce a sense of agency and relational connectedness. Outcomes such as emotional hope, psychological well-being, and civic engagement may therefore not only result from existential hope but also help sustain and deepen it over time. Thus, existential hope is best understood as a dynamic, self-reinforcing system in which emotional, moral, relational, and behavioral processes continuously interact.

It is also important to underscore that, although the present framework is primarily situated within psychology, hope has long been studied across multiple disciplines, including philosophy, theology, sociology, education, and cultural studies. These traditions have contributed important insights into the moral, relational, cultural, and societal dimensions of hope that extend beyond individual psychological processes. Furthermore, growing cross-cultural research suggests that understandings and expressions of hope may vary across cultural contexts, reflecting different conceptions of selfhood, community, spirituality, and the future (Krafft et al., 2023). The present framework should therefore be understood as a psychologically grounded conceptualization of existential hope that is informed by multidisciplinary perspectives and that may benefit from further dialogue with interdisciplinary and cross-cultural perspectives in future research.

### 5.1 Future directions for empirical research

An important first direction for future research concerns the development of a psychometrically sound measure of existential hope. Scale development should proceed systematically, beginning with item generation grounded in theories about existential hope and its components. Both deductive (theory-driven) and inductive (qualitative interview-based) approaches may be valuable to ensure content validity, particularly in populations facing existential disruption (e.g., climate anxiety, chronic illness, cultural marginalization). Factor analytic approaches can then be conducted to establish dimensionality, followed by tests of internal consistency, test–retest reliability, convergent validity (e.g., with meaning in life, psychological flexibility, collective efficacy), and discriminant validity (e.g., from optimism, goal-related hope, and denial-based coping). Establishing measurement invariance across age groups, cultural contexts, and types of existential threat will be crucial to determine whether existential hope functions as a generalizable construct or is indeed context-specific.

A second research direction concerns empirical testing of the proposed integrative framework as a whole. Using a newly developed existential hope scale alongside established measures of perceived radical uncertainty, the four adaptive processes, and proposed outcomes (emotional hope, psychological well-being, and civic engagement), researchers could examine the structural relations among these components. Structural equation modeling (SEM) would be particularly well suited for this purpose, as it would allow for the simultaneous estimation of latent constructs, mediation pathways, and indirect effects (Bollen, 1989). SEM could test whether the adaptive processes and components of existential hope mediate the relationship between perceived radical uncertainty or threat and the relevant outcomes. Competing models could be compared to evaluate model fit. In addition to this latent variable approach, psychometric network analysis (Epskamp et al., 2018a) could provide a complementary perspective by modeling the contextual factors, adaptive processes, existential hope, and outcomes as a system of directly interacting elements. Such analyses could identify central components, bridge nodes linking adaptive processes to outcomes, and clusters of mutually reinforcing processes. Longitudinal designs would be especially valuable for assessing the proposed recursive and feedback dynamics. For example, random-intercept cross-lagged panel models could examine reciprocal influences between adaptive processes, existential hope, and outcomes over time (Hamaker et al., 2015. Temporal network models could illuminate addional dynamic feedback relations among individual components of the system (Epskamp et al., 2018b).

A third research direction concerns the development and empirical testing of interventions. Although it is beyond the scope of this paper to discuss intervention strategies in detail, several promising approaches can be outlined. The integrative framework proposed here provides a conceptual basis for developing interventions that promote existential hope among individuals facing perceived existential threat and radical uncertainty. In addition to promoting existential trust, meaning, and agency, such interventions should explicitly target the four underlying adaptive processes.

As previously mentioned, an established therapeutic approach that directly addresses embracing uncertainty and value articulation is Acceptance and Commitment Therapy (ACT). ACT is a form of cognitive behavioral therapy aimed at increasing psychological flexibility (Hayes et al., 2012). Psychological flexibility refers to the capacity to remain in contact with difficult thoughts and emotions while choosing actions aligned with personally endorsed values. ACT is grounded in the premise that unwanted emotions and thoughts are an inevitable part of life, and that experiential avoidance often intensifies suffering. Through experiential exercises, individuals learn to accept distress associated with radical uncertainty and existential threat while committing to meaningful action. ACT also offers a wide range of value-clarification exercises that support the articulation of personally meaningful directions (Hayes et al., 2012). Substantial evidence demonstrates the effectiveness of ACT in reducing psychological symptoms such as anxiety and depression and enhancing well-being in both clinical and non-clinical populations (e.g., Ferreira et al., 2022; Bohlmeijer et al., 2015; Fledderus et al., 2012). Closely related are compassion-based interventions (Neff, 2023a; Kirby, 2025). Particularly relevant is the concept of fierce compassion, which refers to the protective and action-oriented dimension of compassion (Neff, 2023b). Fierce compassion involves setting boundaries, confronting injustice, and engaging in courageous action rooted in care. Rather than denying suffering or becoming overwhelmed by it, fierce compassion acknowledges pain while affirming that change is both possible and worthwhile. By integrating clarity about reality with committed action, it nurtures a form of hope that is not naïve optimism but a resilient orientation toward constructive response in the face of existential threat (Neff, 2023b).

The process of futuring may be strengthened through interventions that cultivate rigorous imagination and the exploration of multiple future scenarios (Damhof and Gulmans, 2023; Sools and Mooren, 2012). Structured narrative and future-oriented exercises can help individuals and groups articulate desired futures and experiment imaginatively with alternative pathways toward them. More broadly, (existential) positive psychology interventions (PPIs) have been proposed as a promising avenue for promoting existential hope (Malboeuf-Hurtubise et al., 2024; Wong, 2020). PPIs are grounded in evidence-based theories of positive functioning and target self-determined motivation (agency), positive emotions and relationships, and meaning creation in the context of suffering. For example, interventions focused on savoring and gratitude may help individuals cultivate more balanced attentional patterns (Bohlmeijer et al., 2021; Ciarrochi et al., 2022). Indeed, as perceived existential threat can narrow attention toward negative events and future dangers, thereby amplifying distress and worry, savoring and gratitude practices could promote awareness and amplification of positive experiences, supporting emotional resilience and psychological resources (Watkins, 2014). Another example of a promising intervention is photovoice (Malboeuf-Hurtubise et al., 2024), a participatory form of art-based practice that fosters personal expression through photography (Greene et al., 2016). Photovoice has been used to stimulate civic engagement, moral reflection, and social identity development (Strack et al., 2004), thereby linking meaning-making to collective orientation.

Overall, interventions aimed at promoting existential hope must adopt a balanced approach. On the one hand, they should acknowledge and validate the distress arising from radical uncertainty and existential threat. On the other hand, they should actively cultivate trust, meaning, and agency grounded in personal values, envisioned futures, and relational trust. Group-based formats may be particularly valuable, as they provide safe relational spaces in which participants can co-construct meaning, common humanity can be experienced and relational orienting can develop (Neff, 2023a; Malboeuf-Hurtubise et al., 2024).

### 5.2 Conclusion

In desperate times, it can feel as if our very existence is at stake, prompting a courageous reimagining and a renewed commitment to futures that transcend the constraints of an unsettling present. Existential hope is an adaptive response to radical uncertainty and to perceived existential threats. It does not deny suffering, nor does it rely on naïve optimism. Instead, it involves sustaining trust, meaning, and agency when the future cannot be clearly defined or is threatening.To our knowledge, this is the first paper to articulate an integrative psychological framework of existential hope, its underlying adaptive processes, and its potential outcomes. By clarifying its conceptual structure, identifying measurable components, and outlining directions for empirical research and intervention development, this framework provides a foundation for systematic investigation. In a world increasingly characterized by ecological, social, and existential disruption, cultivating existential hope may not only enhance individual well-being but also strengthen collective capacities for constructive engagement and societal transformation.
